# Semi-automatic segmentation of myocardium at risk in T2-weighted cardiovascular magnetic resonance

**DOI:** 10.1186/1532-429X-14-10

**Published:** 2012-01-31

**Authors:** Jane Sjögren, Joey FA Ubachs, Henrik Engblom, Marcus Carlsson, Håkan Arheden, Einar Heiberg

**Affiliations:** 1 Department of Clinical Physiology, Skåne University Hospital, Lund University, Lund, Sweden; 2 Department of Numerical Analysis, Centre for Mathematical Sciences, Lund University, Lund, Sweden

## Abstract

**Background:**

T2-weighted cardiovascular magnetic resonance (CMR) has been shown to be a promising technique for determination of ischemic myocardium, referred to as myocardium at risk (MaR), after an acute coronary event. Quantification of MaR in T2-weighted CMR has been proposed to be performed by manual delineation or the threshold methods of two standard deviations from remote (2SD), full width half maximum intensity (FWHM) or Otsu. However, manual delineation is subjective and threshold methods have inherent limitations related to threshold definition and lack of *a priori *information about cardiac anatomy and physiology. Therefore, the aim of this study was to develop an automatic segmentation algorithm for quantification of MaR using anatomical *a priori *information.

**Methods:**

Forty-seven patients with first-time acute ST-elevation myocardial infarction underwent T2-weighted CMR within 1 week after admission. Endocardial and epicardial borders of the left ventricle, as well as the hyper enhanced MaR regions were manually delineated by experienced observers and used as reference method. A new automatic segmentation algorithm, called Segment MaR, defines the MaR region as the continuous region most probable of being MaR, by estimating the intensities of normal myocardium and MaR with an expectation maximization algorithm and restricting the MaR region by an *a priori *model of the maximal extent for the user defined culprit artery. The segmentation by Segment MaR was compared against inter observer variability of manual delineation and the threshold methods of 2SD, FWHM and Otsu.

**Results:**

MaR was 32.9 ± 10.9% of left ventricular mass (LVM) when assessed by the reference observer and 31.0 ± 8.8% of LVM assessed by Segment MaR. The bias and correlation was, -1.9 ± 6.4% of LVM, R = 0.81 (p < 0.001) for Segment MaR, -2.3 ± 4.9%, R = 0.91 (p < 0.001) for inter observer variability of manual delineation, -7.7 ± 11.4%, R = 0.38 (p = 0.008) for 2SD, -21.0 ± 9.9%, R = 0.41 (p = 0.004) for FWHM, and 5.3 ± 9.6%, R = 0.47 (p < 0.001) for Otsu.

**Conclusions:**

There is a good agreement between automatic Segment MaR and manually assessed MaR in T2-weighted CMR. Thus, the proposed algorithm seems to be a promising, objective method for standardized MaR quantification in T2-weighted CMR.

## Background

Myocardium at risk (MaR) is defined as the ischemic myocardium during coronary artery occlusion and is the region that will be subject to infarction if the blood flow is not restored. Myocardium at risk can be measured using T2-weighted cardiovascular magnetic resonance(CMR) [1] due to the myocardial edema occurring in the ischemic myocardium [2,3] up to one week after percutaneous coronary intervention (PCI) [4]. By determining MaR using T2-weighted CMR and myocardial infarction (MI) size using late gadolinium enhancement (LGE), the efficacy of reperfusion therapy can be assessed as myocardial salvage in a single CMR session.

In the event of an acute coronary occlusion, a single artery is usually affected. As a consequence of the occlusion, transmural ischemia occurs within the affected coronary artery's perfusion territory [5,6]. The myocardium subjected to ischemia becomes edematous and shows an increased signal intensity in T2-weighted CMR compared to non-ischemic myocardium[7]. Several techniques have been proposed for quantitative assessment of MaR in T2-weighted CMR, such as manual delineation [4], and threshold methods of two standard deviations (2SD) from remote [5,8], full width half maximum (FWHM) intensity [9] and Otsu [10]. Human observers delineating MaR take into account both regional intensity differences and *a priori *knowledge on perfusion territories and transmurality, which may improve accuracy of MaR quantification. However, manual delineation is subjective and time consuming. Semi-automatic and automatic threshold methods such as 2SD, FWHM and Otsu have been proposed as more objective methods. A more advanced automatic algorithm for quantification of edema in T2-weighted CMR has recently been developed by Johnstone et al [11]. Their algorithm shows promising result for an automatic segmentation approach of edema and thereby MaR in T2-weighted CMR by incorporating regional analysis. However, neither the threshold methods, (2SD, FWHM and Otsu), nor the algorithm by Johnstone et al. uses *a priori *knowledge on the appearance of MaR and the cardiac anatomy, which is considered when performing manual delineations.

Therefore, the aim of this study was to develop an automatic segmentation algorithm for quantification of MaR in T2-weighted CMR images which uses *a priori *knowledge on the appearance of MaR and cardiac anatomy.

## Methods

### Study Population

The study was approved by the local ethics committee and all patients gave their written informed consent. Forty seven patients (age 60.3 ± 9.8 years, range 39 - 83, 39 males) with first-time acute ST-elevation myocardial infarction (STEMI) due to a single occluded coronary artery confirmed by angiography were prospectively included in the study. All patients were treated with primary percutaneous coronary intervention (PCI) with coronary stenting, resulting in TIMI grade 3 flow in the culprit artery.

### CMR imaging

Within a week after admission patients were imaged in the supine position using either a 1.5 T system (Magnetom Vision, Siemens, Erlangen, Germany) with a CP body array coil or a 1.5 T system (Philips Intera CV or Achieva, Philips, Best, the Netherlands) with a cardiac synergy coil. Initial scout images were acquired to locate the heart, and a T2-weighted triple inversion turbo spin echo sequence (STIR) was employed to depict the myocardium at risk. T2-weighted CMR images were acquired in the short-axis view, covering the left ventricle from the base to apex. Imaging parameters were: echo time 43 ms (Siemens) or 100 ms (Philips); repetition time 2 heart beats; number of averages 2; inversion time 180 ms; typical image resolution 1.5 × 1.5 mm (Siemens) or 1.4 × 1.7 mm reconstructed to 0.7 × 0.7 mm (Philips); slice thickness 8 mm with a typical slice gap of 2 mm. When acquiring images with the cardiac synergy coil no parallel imaging was performed (SENSE = 1).

### Image analysis

The MaR was manually delineated according to the method previously described by Carlsson et al [4]. In short, endocardial and epicardial borders of the LV were traced in all short-axis slices by an experienced observer and the papillaries were excluded from the myocardium. Regions of hyper enhanced myocardium was manually delineated as myocardium at risk (MaR) by an experienced observer and expressed as percent of left ventricular mass (LVM). Hypo-intense myocardium within the area of increased signal intensity was regarded as microvascular obstruction [12] and was included in the MaR.

The new segmentation algorithm, called Segment MaR, was implemented in the freely available cardiac image analysis software Segment (http://segment.heiberg.se) [13] and will be made available at time of publication. Segment was also used for manual delineation and implementation of the threshold methods (2SD, FWHM and Otsu).

### Automatic segmentation algorithm, Segment MaR

The automatic segmentation algorithm, Segment MaR, defines the MaR within the manually delineated left ventricular myocardium based on the culprit artery defined by the user. The MaR region is defined as a continuous region which has a higher probability of being MaR compared to normal myocardium, based on the signal intensity, and fulfills *a priori *criteria for MaR regarding transmurality, shape, size and extent within the perfusion territory of the culprit artery.

**Figure 1:**
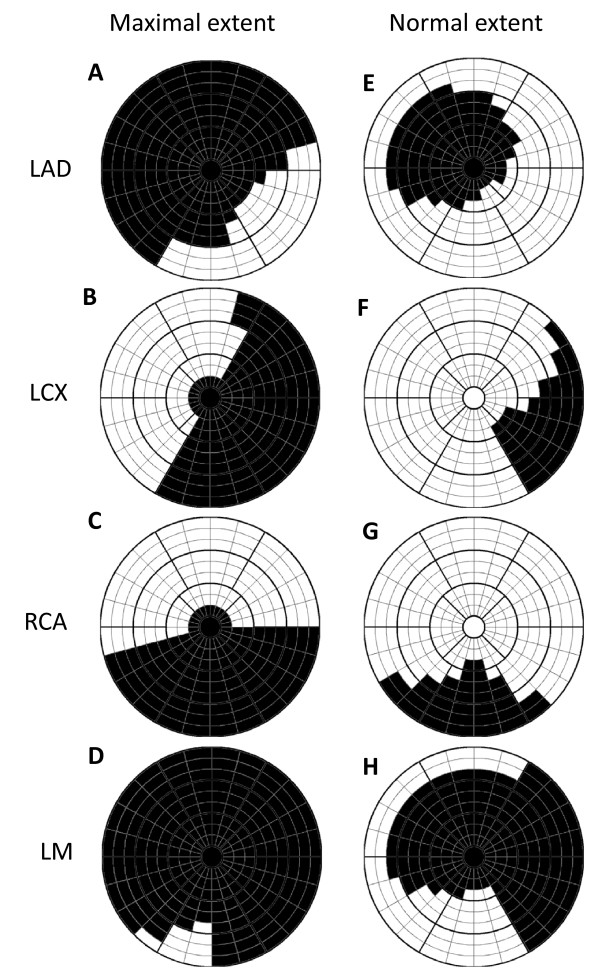
**Maximal and normal extent model**. Bulls-eye representation of maximal extent model (left column) and normal extent model (right column) for the perfusion territories of left anterior descending artery (LAD), left circumflex artery (LCx), right coronary artery (RCA), and left main artery (LM). Models for LAD, LCX and RCA were defined in consensus by three experienced observers in an extended 17-segment AHA model and models for LM were defined from the models of LAD, LCX and RCA. The 17-segment model is extended to three slices in each of the basal, mid-ventricular and apical zones and 24 sectors in each slice. Black sectors are included in the maximal and normal extent model, respectively. The septal part of the left ventricle is represented in the left of the bulls-eye plot, the lateral part in the right, anterior part in the top, inferior part in the bottom, the apical slices in the center and the basal slices in the outer part of the bulls-eye plot.

Figure 1 shows a model of normal and maximal extent for the perfusion territories of each coronary artery as defined in consensus by three experienced observers from their combined experience of CMR and SPECT. Normal and maximal extent models were defined by each observer and discussed until consensus was reached for left anterior descending artery (LAD), left circumflex artery (LCx) and right coronary artery (RCA). The models for left main artery (LM) were defined from the models of LAD, LCx and RCA. The extent was defined in the 17 segment AHA model [14] which was extended to 24 sectors circumferentially and 3 slices in each of the basal, mid-ventricular and apical parts of the left ventricle. The maximal and normal extent model of the user defined culprit artery is used as *a priori *information in the algorithm.

**Figure 2:**
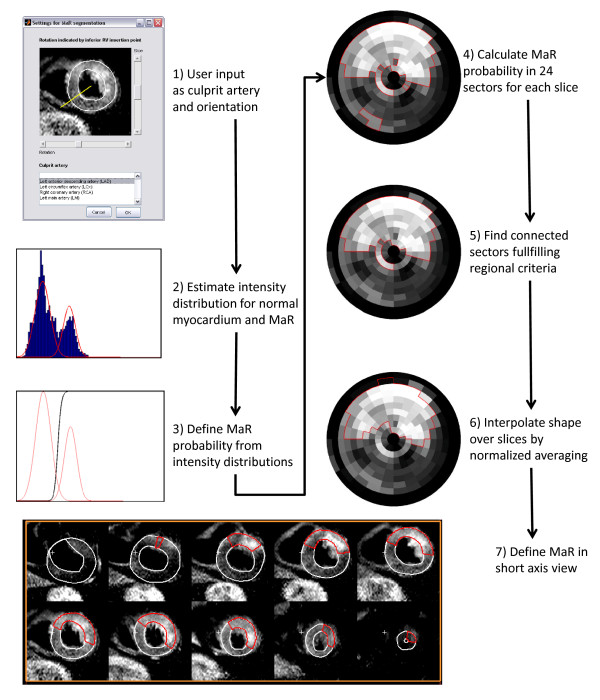
**Flow chart for automatic segmentation algorithm**. Flow chart of the automatic Segment MaR algorithm from user input to segmentation result. Step 1) shows the user interface for user input, step 2) shows the intensity histogram for a short axis slice and the estimated intensity distributions for normal myocardium and MaR, step 3) shows the MaR probability function in black and the estimated intensity distributions in red, step 4) shows the sector based bulls eye plot of MaR probability, with bright colors indicating high probability of MaR, in step 5) the MaR region of connected sectors fulfilling the regional criteria is marked in red and in step 6) the interpolated shape of the MaR region is marked in red and finally in step 7) the MaR region is shown in red in a short axis view.

The Segment MaR algorithm can be divided into 7 steps (Figure 2)

1) User input as culprit artery and orientation

2) Estimate intensity distribution for normal myocardium and MaR

3) Define MaR probability from intensity distributions

4) Calculate MaR probability in 24 sectors for each short axis slice

5) Find region of connected sectors with high probability of MaR which fulfills regional criteria

6) Interpolate shape of MaR region over slices by normalized averaging

7) Define MaR region in the short-axis view from sector based segmentation

In step 1) the user defines the culprit artery as either, LAD, LCx, RCA or LM based on the overall appearance of the hyperenhanced region and indicates the orientation of the heart by indicating the inferior insertion point for the right ventricle (Figure 2:1).

In step 2) the intensity distribution for normal myocardium and MaR is estimated from the intensity histogram by an expectation maximization (EM) algorithm slice by slice [15]. The intensity distributions were analyzed slice by slice since the intensity can vary between slices in T2-weighted CMR images. The EM-algorithm estimates the mean and standard deviation of the intensity distributions for MaR and normal myocardium by refining an initial estimation. The initial estimation of mean and standard deviation for MaR is calculated from the intensities within the normal extent model for the culprit artery and for normal myocardium calculated from the intensities outside the maximal extent model for the culprit artery. The intensity distributions for MaR and normal myocardium are assumed to be Gaussian and are thereby defined by their mean and standard deviation as estimated by the EM-algorithm. Figure 2:2 shows the intensity histogram of the myocardium and the estimated Gaussian intensity distributions for normal myocardium and MaR.

In step 3) a MaR probability is defined from the intensity distributions for normal myocardium and MaR. The MaR probability is defined from the Bayesian probability of MaR given that the intensity is either MaR or normal myocardium by dividing the Gaussian intensity distribution for MaR by the sum of the Gaussian distribution for MaR and normal myocardium. Thus, the probability function is in the range from 0 to 1 and values above 0.5 indicate higher probability of MaR than normal myocardium. A probability function and its corresponding Gaussian distributions are shown in Figure 2:3.

In step 4) the MaR probability is calculated for 24 sectors in each short axis slice. The probability function in step 3) is defined for each short axis slice and mapped to each pixel in the slice. The probability value is then averaged for 24 sectors in each slice resulting in a sector-based MaR probability. The sector-based MaR probability is shown in Figure 2:4 where bright colors indicate high probability of MaR and the red border indicates sectors with a probability value above 0.5.

In step 5) a region of connected sectors with a high probability for MaR which fulfills regional criteria is identified. The criteria to be fulfilled is a) sectors with a probability value above 0.5 should be connected to its nearest neighboring sector within the slice or in an adjacent slice in a 4-neighbourhood to constitute a region, b) sectors should be localized within the maximal extent model for the culprit artery, c) in the slices with outflow tract only sectors on the anterior side of the outflow tract is considered MaR for LAD and LM and only sectors on the inferior side for LCx and RCA. Finally, the MaR probability of each region is calculated by summing the probability value of being MaR for each pixel within the region and summing the probability of being normal i.e. 1 minus the probability of being MaR for each pixel outside the region. If multiple connected regions are found, the region with the highest probability is chosen and the other regions are eliminated from the MaR region. The outer boundary of the new MaR region is indicated with a red border in Figure 2:5.

In step 6), the shape of the MaR region is refined by interpolating the outer boundary of the MaR region over slices by normalized averaging [16].The normalized averaging interpolates the outer boundary by using certainty values for the outer boundary of each slice and a narrow kernel with width of 3 slices in both apical and basal direction. The certainty value is lowered if a) the region is close to the maximal extent model, b) if the difference in extent deviates from normal difference between slices and c) if the intensity appearance does not match the boundary of the MaR region. The certainty based on closeness to the maximal extent, a), is calculated by a linear function from one to zero between the normal and maximal extent model in each slice. The certainty based on difference in extent between slices, b), is calculated from a Gaussian function with standard deviation of two sectors and a mean of increasing two sectors from base to apex for LAD and LM respectively a mean of decreasing one sector for RCA and LCx. The certainty based on intensity appearance, c), was calculated as the MaR probability, as defined in step 5, for each slice. The new boundary defined by the normalized averaging is interpolated over slices to give a smooth appearance of the MaR region (Figure 2:6).

In step 7) the MaR region is defined in a short axis view from the sector based MaR region. This is done by defining all pixels within a sector as MaR if the sector is within the outer boundary of the sector based MaR region (Figure 2:7).

### Comparison to other segmentation methods

The new automatic segmentation method, Segment MaR, was compared to inter observer variability of manual delineation and three threshold methods, 2 standard deviations from remote (2SD), full width half maximum intensity (FWHM) and Otsu. All methods used the same manual delineation of endocardium and epicardium and were applied slice by slice. A slice by slice approach was chosen since the intensity varies between slices in T2-weighted images.

A second experienced and independent observer manually delineated MaR in all subjects for inter observer analysis. The 2SD threshold method estimates an intensity threshold from a remote region as the mean plus two standard deviations of the intensity within the remote region. The remote region was defined as the region outside the maximal extent model of the culprit artery, indicated by the white sectors in the maximal extent model in Figure 1. The FWHM threshold method [17] estimates an intensity threshold from a remote region as midway between the mean intensity within the remote region and the maximal intensity within the myocardium. The remote region was defined in the same way as for 2SD. The threshold method of Otsu [18] estimates the intensity threshold from the histogram of all intensities to get minimal variance both above and below the threshold. The intensity threshold was calculated and applied slice by slice for all three threshold methods.

### Statistical analysis

The quantification of MaR by the automatic Segment MaR algorithm, manual second observer delineation, the threshold methods of 2SD, FWHM and Otsu were all compared against the reference observer using Bland-Altman bias (mean ± standard deviation), paired t-test and linear regression analysis (correlation coefficient and p-value). Regional agreement to manual delineation by the reference observer was evaluated by calculating the Dice similarity coefficient (DSC) [19], which can be derived from the kappa statistics for the classification of pixels [20]. The DSC is calculated as two times the volume of the intersection of the MaR regions divided by the sum of the volumes of the two MaR regions. The DSC is therefore 0 if the regions do not overlap and 1 if the regions overlap perfectly. The DSC was calculated against the reference observer, for Segment MaR, second observer delineation, 2SD, FWHM and Otsu, for each patient and expressed as mean ± standard deviation.

## Results

**Figure 3:**
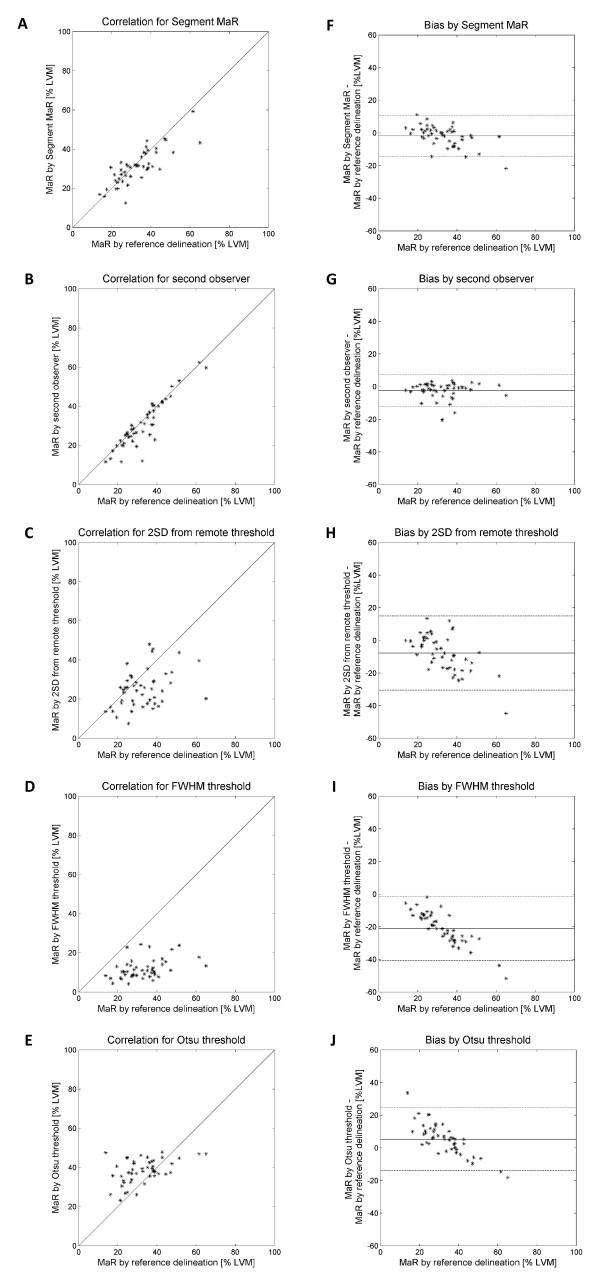
**Correlation and Bland-Altman plot for all five segmentation methods**. Correlation of MaR as % of LVM (panel A-E) and Bland-Altman plot of MaR bias as % of LVM (panel F-J) against the reference delineation for the automatic Segment MaR algorithm (panel A, F), second observer delineation (panel B, G), the threshold methods of two standard deviations from remote threshold (2SD)(panel C, H), full width half maximum (FWHM)(panel D, I) and Otsu (panel E,J).

**Figure 4:**
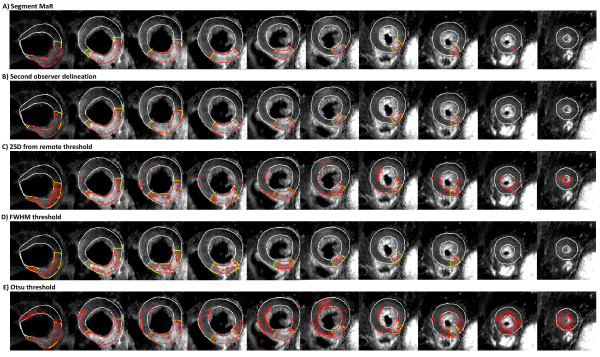
**Typical segmentation result for all five segmentation methods**. Typical MaR segmentation shown in red for the automatic segmentation Segment MaR (panel A), second observer delineation (panel B), the threshold methods of two standard deviations from remote (2SD) (panel C), full width half maximum (FWHM) (panel D) and Otsu (panel E), compared to manual delineation by the reference observer, shown in yellow, all within the manual delineation of myocardial borders (shown in white). The same patient, short-axis slices, manual delineation of myocardial borders and manual reference delineation of MaR is used for all methods and shown from most basal slice in the left of the panel to the most apical slice in the right of the panel. Note the continuous appearance of the segmentation for Segment MaR and manual delineation by the reference and second observer compared to the threshold methods of 2SD, FWHM and Otsu.

MaR assessed by the reference observer was 32.9 ± 10.9% of LVM and MaR assessed by Segment MaR was 31.0 ± 8.8%. There was a strong correlation, R = 0.81, p < 0.001, and low bias, -1.9 ± 6.4% of LVM, p = 0.047, when Segment MaR was compared to the reference delineation of MaR (Table 1, Figure 3). The inter observer variability of manual delineation as the bias between reference and second observer was -2.3 ± 4.9% of LVM. The bias for Segment MaR was lower than for the threshold methods of 2SD, FWHM and Otsu, -7.7 ± 11.4% of LVM, -21.0 ± 9.9% of LVM and 5.3 ± 9.6% of LVM, respectively (Table 1). Furthermore there was a good regional agreement between Segment MaR and the manual reference delineation, DSC = 0.85 ± 0.07 (Table 1). In Figure 4 typical segmentations for all five methods are shown in the same patient and compared to manual delineation by the reference observer. For Segment MaR and manual delineation by the reference and second observer, the MaR region is continuous whereas the segmentation by the threshold methods of 2SD, FWHM and Otsu consist of multiple regions of hyperenhanced myocardium.

**Table 1:** Results for all five segmentation methods compared to reference delineation

	MaR bias		Regression		
	[% of LVM]	p-value	R-value	p-value	DSC
Segment MaR	-1.9 ± 6.4	0.047	0.81	<0.001	0.85 ± 0.07

Second observer delineation	-2.3 ± 4.9	0.003	0.91	<0.001	0.90 ± 0.08

2SD threshold	-7.7 ± 11.4	<0.001	0.38	0.008	0.69 ± 0.14

FWHM threshold	-21.0 ± 9.9	<0.001	0.41	0.004	0.46 ± 0.14

Otsu threshold	5.3 ± 9.6	<0.001	0.47	<0.001	0.68 ± 0.10

MaR-Myocardium at risk, DSC-Dice similarity coefficient, 2SD-two standard deviations from remote, FWHM-full width half maximum intensity

**Figure 5:**
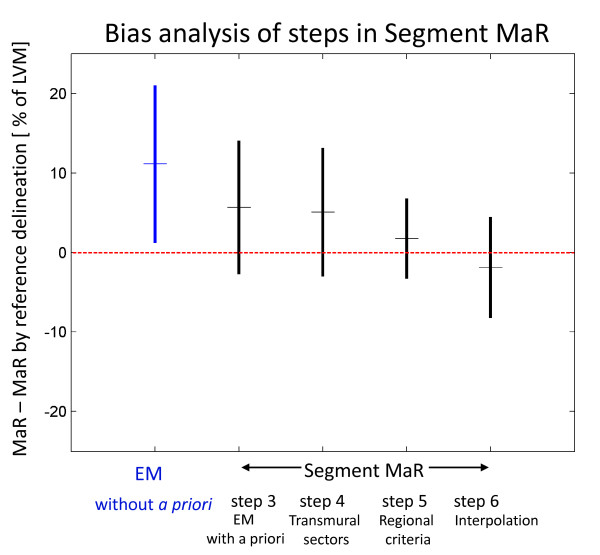
**Bias analysis of steps in Segment MaR**. Bias as mean ± standard deviation to manual delineation for EM-algorithm without *a priori *information, indicated in blue, and for steps 3, 4, 5 and 6 of Segment MaR, indicated in black. Mean is indicated with a horizontal line on the middle of the vertical line which indicates mean ± standard deviation. The red dashed line shows zero bias to manual delineation by reference observer. EM-algorithm without *a priori *information is the baseline. Step 3 is EM algorithm with *a priori *information, step 4 introduces transmural sectors, step 5 uses *a priori *regional criteria and step 6 uses interpolation to get physiological and smooth appearance. Note how the bias and standard deviation is decreased by utilizing *a priori *information in step 3 and 5.

In order to analyze the added value of each step in the Segment MaR algorithm (Figure 2) the bias to manual delineation by the reference observer was calculated after steps 3, 4, 5 and 6. As a base-line, bias was calculated for segmentation by the EM-algorithm without *a priori *information. The results are shown as mean ± standard deviation in Figure 5.

In eight of the forty-seven patients multiple hyperenhanced regions were detected in step 5 of the algorithm. In seven out of those the same region as by manual delineation was identified. In the one case, a dark artifact divided the MaR region into two disconnected regions, resulting in the Segment MaR algorithm only identifying one of these and subsequently underestimating the MaR region.

## Discussion

This study has presented an automatic segmentation algorithm for quantification of MaR from T2-weighted CMR, based on the EM-algorithm and *a priori *information on normal and maximal perfusion territories for the culprit artery. Compared to manual delineation by a reference observer, the new algorithm, Segment MaR, performed better than previously suggested threshold methods (2SD, FWHM and Otsu). Quantitative bias and regional agreement for Segment MaR were similar to inter observer variability of manual delineation.

The new automatic segmentation algorithm, Segment MaR, estimates an intensity based probability of MaR from all intensity information in each short axis slice by an EM-algorithm. The use of all intensity information may make the estimate more robust to noise, artifacts and variation in signal homogeneity. The use of *a priori *information in the EM-algorithm showed an added value compared to using the EM-algorithm without *a priori *information in the bias analysis (Figure 5). The constraint of the extent by a maximal extent model eliminates artifacts located outside the perfusion territory and together with the use of only one region of connected sectors step 5 showed an added value in the bias analysis. Small non-transmural artifacts within the perfusion territory of the culprit artery may also be less likely to be considered as MaR by requiring transmural regions from the connected sectors. The bias analysis showed no added value by use of the interpolation although it is needed for a physiological appearance of the MaR region.

In the edema algorithm by Johnstone et al. [11] an EM-algorithm was used to estimate the intensity distributions and a threshold was defined as 2 standard deviations above the mean intensity of normal myocardium. Thereby the threshold is similar to that of 2SD threshold with the exception that the intensity of normal myocardium is defined from the EM-algorithm instead of a remote region. Using a threshold of 2SD does not utilize the intensity information on MaR which has been estimated by the EM-algorithm and thereby the variation in signal intensity may not be taken into consideration. Another difference between Segment MaR and the edema algorithm by Johnstone is that the Segment MaR algorithm is based on 24 sectors in each slice instead of pixel wise segmentation. The pixel-wise segmentation does not consider transmurality and may give better precision on the boundary of MaR. Pixel-wise segmentation may, however, be more sensitive to artifacts. Quantitative results for the edema algorithm, reported by Johnstone, were a bias of 1.1 ± 10.1% of LVM and DSC of 0.50 ± 0.27 to their reference of manual delineation and an aim for future work was set to reach DSC > 0.7 which has been stated to indicate excellent regional agreement by Zijdenbos et al. [20]. In the present study, Segment MaR showed a DSC value of 0.85 ± 0.07 and bias of -1.9 ± 6.4% of LVM compared to the manual delineation of the reference observer. Note-able is also the difference in DSC for inter observer variability of manual delineation which was 0.72 ± 0.14 in the study by Johnstone compared to 0.90 ± 0.08 in the present study. This may indicate that the regional agreement may be lower and the quantitative variability of manual delineation may be larger between observers than reported in the present study and thereby the use of an automatic algorithm such as Segment MaR, which has a low bias and high regional agreement to manual delineation, may decrease the inter observer variability.

The weak correlation to manual delineation by the reference observer for the threshold methods of 2SD, FWHM and Otsu may be explained by the fact that the methods are solely based on a threshold and by weaknesses in defining the threshold for the different methods. Using a fixed number of standard deviations as in 2SD does not account for the variability of intensity for MaR and more importantly the contrast in T2-weighted CMR is lower than for other CMR [21]. The threshold by FWHM may be sensitive to artifacts since the brightest pixel intensity is used to find the threshold. Both 2SD and FWHM are sensitive to the definition of the remote region which is currently not standardized. The remote region in this study was automatically defined as the myocardium outside the maximal extent model of the culprit artery. This definition of the remote myocardium was chosen to obtain an objective and standardized representation of normal myocardium. This strategy may, however, result in artifacts being included in the remote myocardium. An overestimation of the threshold may for 2SD be caused by the large standard deviation of the intensity within the remote region. The border zone between normal myocardium and remote myocardium influences the remote myocardium proportionally more for larger MaR regions which may result in a overestimation of the threshold and subsequent underestimation of the MaR region and thus explain the trend seen in bias for 2SD (Figure 3). For FWHM an overestimation of the threshold may be caused by artifacts within the myocardium and the remote region. Bright artifacts may result in a threshold which only identifies the artifact as the MaR region and this can explain the trend in bias seen for FWHM since it will result in a larger underestimation for larger MaR regions. The threshold defined by Otsu does not depend on any remote region but may instead be unstable in the definition of the threshold since it assumes that an optimal threshold should be found in each short axis slice. This implies that both MaR and normal myocardium should be present in each slice, which is not the case in most patients as for example in the basal slices of an LAD occlusion or apical slices of an RCA occlusion. The Otsu threshold may thereby overestimate the MaR region in slices lacking MaR and underestimate MaR in slices lacking normal myocardium. This may explain the large overestimation for small MaR regions and large underestimation for large MaR regions. The EM-algorithm used in Segment MaR also assumes two intensity distributions as in the threshold method of Otsu but is accompanied by *a priori *information both as initialization to the EM-algorithm and in the post processing of finding connected sectors.

Manual monitoring and possibly manual corrections are as for all automatic segmentation algorithms needed for research and clinical use. The use of Segment MaR may, however, decrease the degree of variability introduced by the subjectivity of manual delineation since the Segment MaR algorithm showed a low bias and high correlation to manual delineation regarding quantitative assessment and an excellent regional agreement according to DSC.

One limitation in this study is the lack of ground truth for in vivo quantification of MaR. Manual delineation according to the methodology used by Carlsson et al. [4] when validating T2-weighted CMR for MaR to SPECT was chosen as reference method and in this study there was a good inter observer agreement. Due to the limited number of patients in the study it was not possible to use a separate training and test set and the parameters in the automatic Segment MaR algorithm could not be optimized. The Segment MaR algorithm has not been specifically designed for the imaging systems of Philips and Siemens. Further research is suggested to investigate the performance of the algorithm in a larger cohort of patients and possibly optimize and improve the algorithm for specific imaging systems.

## Conclusions

In this study, an automatic segmentation algorithm, called Segment MaR, for quantification of myocardium at risk (MaR) in T2-weighted CMR has been presented and showed to have a good agreement to manual delineation. Both the quantitative and regional agreement to manual delineation was better for Segment MaR than for the threshold methods of two standard deviations from remote, full width half maximum intensity and Otsu. The Segment MaR algorithm seems to be a promising, objective method for standardized measurement of MaR in T2-weighted CMR.

## Competing interests

EH is the founder of Medviso AB, Lund, Sweden, which sells a commercial version of Segment. JS is employed by Medviso AB on a part-time basis. The other authors declare that they have no competing interests.

## Authors' contributions

All authors contributed to concept and design of the study, input to the development of an automatic segmentation algorithm and revised the manuscript. All authors read and approved the final manuscript. JS developed and implemented Segment MaR, implemented the threshold methods, analyzed and interpreted segmentation results, and drafted the manuscript. JU included patients to the study and performed manual delineation of the left ventricle. HE performed manual delineation of MaR as second observer, defined maximal and normal extent models, and analyzed and interpreted data. MC defined maximal and normal extent models, and analyzed and interpreted data. HA performed manual delineation of MaR as reference observer, defined maximal and normal extent models and conceived the study. EH analyzed and interpreted data and conceived the study.
